# Safety Surveillance of Mass Praziquantel and Albendazole Co-Administration in School Children from Southern Ethiopia: An Active Cohort Event Monitoring

**DOI:** 10.3390/jcm11216300

**Published:** 2022-10-26

**Authors:** Tigist Dires Gebreyesus, Eyasu Makonnen, Tafesse Tadele, Habtamu Gashaw, Workagegnew Degefe, Heran Gerba, Birkneh Tilahun Tadesse, Parthasarathi Gurumurthy, Eleni Aklillu

**Affiliations:** 1 Division of Clinical Pharmacology, Department of Laboratory Medicine, Karolinska Institutet, Karolinska University Hospital Huddinge, 14186 Stockholm, Sweden; 2 Ethiopian Food and Drug Authority, Addis Ababa P.O. Box 5681, Ethiopia; 3 Center for Innovative Drug Development and Therapeutic Trials for Africa, College of Health Sciences, Addis Ababa University, Addis Ababa P.O. Box 9086, Ethiopia; 4 Departments of Pharmacology and Clinical Pharmacy, College of Health Sciences, Addis Ababa University, Addis Ababa P.O Box 9086, Ethiopia; 5 College of Medicine and Health Sciences, Hawassa University, Hawassa P.O. Box 1560, Ethiopia; 6 Pharmacovigilance and Clinical Trials, Botswana Medicines Regulatory Authority, Gaborone P.O. Box 505155, Botswana

**Keywords:** safety surveillance, pharmacovigilance, praziquantel, albendazole, school children, preventive chemotherapy, cohort event monitoring, schistosomiasis, STH, Ethiopia, drug safety

## Abstract

Preventive chemotherapy (PC) with praziquantel and albendazole co-administration to all at-risk populations is the global intervention strategy to eliminate schistosomiasis and soil-transmitted helminth (STH) from being public health problems. Due to weak pharmacovigilance systems, safety monitoring during a mass drug administration (MDA) is lacking, especially in sub-Saharan Africa. We conducted large-scale active safety surveillance to identify the incidence, types, severity, and associated risk factors of adverse events (AEs) following praziquantel and albendazole MDA in 5848 school children (5–15 years old). Before MDA, 1484 (25.4%) children were prescreened for *S. mansoni* and STH infections, of whom 71.8% were infected with at least one parasite; 34.5% (512/1484) had *S. mansoni* and 853 (57.5%) had an STH infection. After collecting the baseline socio-demographic, clinical, and medical data, including any pre-existing clinical symptoms, participants received single dose praziquantel and albendazole MDA. Treatment-associated AEs were actively monitored on days 1 and 7 of the MDA. The events reported before and after the MDA were cross-checked and verified to identify MDA-associated AEs. The cumulative incidence of experiencing at least one type of MDA-associated AE was 13.3% (95% CI = 12.5–14.2%); 85.5%, 12.4%, and 1.8% of reported AEs were mild, moderate, and severe, respectively. The proportion of experiencing one, two, or ≥ three types of AEs was 57.7%, 34.1%, and 8.2%, respectively. The cumulative incidence of AEs in *S. mansoni*- and (17.0%) and STH (14.1%)-infected children was significantly higher (*p* < 0.001, χ^2^ = 15.0) than in non-infected children (8.4%). Headache, abdominal pain, vomiting, dizziness, and nausea were the most common AEs. Being female, older age, having *S. mansoni* or STH infection were significant predictors of MDA-associated AEs. In summary, praziquantel and albendazole co-administration is generally safe and tolerable. MDA-associated AEs are mostly mild-to-moderately severe and transient. The finding of few severe AEs and significantly high rates of AEs in helminth-infected children underscores the need to integrate pharmacovigilance in MDA programs, especially in high schistosomiasis and STH endemic areas.

## 1. Introduction

In sub-Saharan Africa (SSA), soil-transmitted helminth (STH) and schistosomiasis are the first and second most prevalent neglected tropical diseases (NTDs), respectively [1]. More than 90% of the global schistosomiasis burden is from SSA. Both STH and schistosomiasis have been associated with different health complications among the chronically infected groups by causing malnutrition, anemia, a poor cognitive function, impaired childhood development, fatigue, and exercise intolerance [2,3,4,5,6]. In Ethiopia, intestinal schistosomiasis, which *Schistosoma mansoni* causes, is endemic in most parts of the country and remains among the main causes of morbidity in the country [6,7,8]. STH is endemic throughout the country.

Due to the long-term health consequences and economic burden in endemic areas, the World Health Organization (WHO) established a global program to eliminate NTDs as a public health problem. Among the interventions recommended by the WHO for schistosomiasis and STH elimination and control is preventive chemotherapy (PC), which is defined as a large scale distribution of anthelmintics at regular intervals without prior screening to all at-risk population groups [9,10,11,12]. School children are the main target for PC using praziquantel and albendazole due to their increased risk of infection [13,14]. In line with the WHO 2012–2020 strategic plan for the control and elimination of NTDs, drug donations for PC through a mass drug administration (MDA) to at-risk population groups has been escalated globally due to increased donor funds and support from pharmaceutical industries [9,15]. This large-scale donation of drugs and funds has resulted in reaching millions of at-risk populations through PC. For instance, 105 million individuals received praziquantel PC against schistosomiasis in 2019. Similarly, 613 million children received PC for the control of STH through the annual deworming program the same year [16].

The WHO has expanded the target population group with a recommendation of annual PC to people older than two years in endemic countries where the prevalence exceeds 10% [17]. The increase in the target population and frequency of PC use will increase the number of individuals who will be exposed to drugs, which will have implications for safety monitoring. Firstly, the large-scale distribution of drugs without a prior screening of individuals for the disease may expose the non-infected ones to unnecessary MDA-related adverse events [17,18]. Secondly, drugs used in PC, like for schistosomiasis and STH, are often co-administered, increasing the risk of rare and unexpected adverse events due to drug–drug interaction and/or overlapping toxicities [19]. Third, most clinical trials on drugs used in an MDA are tested in different population groups than the target population for the MDA. Thus, host-genetic and environmental variations may predispose the target population to the occurrence of rare adverse events. Finally, the drug distribution during PC implementation is mainly conducted by non-healthcare professionals with no or little knowledge of drug-related adverse events, further underscoring the need for integrating safety monitoring (pharmacovigilance) in PC programs.

Adverse events following an MDA might be associated with the disease characteristics (the intensity of infection), participant characteristics (their age and nutritional status), or other factors in the context of drug use (operational errors or coincidences) [20,21,22]. Therefore, the integration of safety monitoring in the NTD control program is crucial for ascertaining the cause of adverse events and taking the necessary preventive and corrective measures. Previous studies have evaluated the safety of praziquantel and/or albendazole [20,21,22,23]. However, these studies had relatively small sample sizes, and some assessed only the safety of praziquantel alone, and the follow-up period was also short. Furthermore, no active surveillance study has evaluated and compared the safety outcome of praziquantel and albendazole between helminth-infected and non-infected children. Hence, safety data on mass praziquantel and albendazole administration, especially in SSA, are scarce.

The pharmacovigilance system in most endemic countries, especially in SSA countries, is weak, and the spontaneous reporting system captures few to no adverse events during an MDA [24,25]. Furthermore, the spontaneous reporting only shows the trend of adverse events and does not help determine the incidence since the denominator (the number of populations who took the drug) is unknown. In settings where the pharmacovigilance system is weak and the implementation of passive surveillance is challenging, other robust safety surveillance mechanisms like active safety monitoring in the form of cohort event monitoring is recommended [26,27]. Therefore, with the aim of identifying the incidence, types, severity, and risk factors of the adverse events associated with mass praziquantel and albendazole co-administration, we conducted a large-scale observational prospective safety surveillance using active cohort event monitoring among school children in selected primary schools in southern Ethiopia.

## 2. Materials and Methods

### 2.1. Study Design, Area, and Population

The study observational prospective active safety surveillance of mass praziquantel and albendazole administration was conducted in six primary schools in the Hawella Tula and Wondo Gennet districts in the southern part of Ethiopia, at approximately 300 km south to Addis Ababa. The former district is located along the shore of lake Hawassa and residents rely on the lake water for their major domestic use. The Wondo Gennet district has various small water sources and most of the community members use these water sources for their consumption purpose. The study participants were school children attending six primary schools in two rural districts located in in Hawella Tula and Wondo Gennet districts. The schools involved from the Hawella Tula district were Bushulo, Kidus Pawulos, Finchawa, and Cheffe primary schools, while those involved from Wondo Gennet district were Wosha and Chukko primary schools. These districts were labeled as high schistosomiasis and STH prevalence districts by the national NTD public program based on the mapping results.

A total of 5848 school children aged 5–15 years from the six schools were enrolled in this study. The sample size for each school was calculated based on the proportion of the student population size. According to the WHO, a sample size of 10,000 gives a 95% probability (confidence) to detect at least three events at a frequency of 1 per 3333 [28,29]. Therefore, based on that estimation, our study sample size of 5848 will detect at least three events at a frequency of 1 per 1949 with a 95% confidence.

### 2.2. Study Enrolment and Baseline Data Collection

This study obtained ethics approval from the Institutional Review Board of the Southern Nations, Nationalities and Peoples Region Health Bureau ethical clearance committee (Ref no 902-6-19/14966) and the Ethiopian national research ethics review committee (Ref no MoSHE//RD/141/9848/20). Before the study’s initiation, permission to conduct the study was obtained from the regional health and education bureaus, zonal and district health, and education offices. Orientation meetings with representatives from the district’s education and health offices, healthcare professionals, schoolteachers, and the school administrator were done to give information about the study. Prior to the enrolment, participants and their parents or legal guardians received information about the study. School children whose parents/guardians gave consent and provided assent to participate (if applicable) were enrolled.

After their enrollment, the children’s baseline socio-demographic data including their age, sex, and, nutritional status using anthropometric data, medical history, any comorbidities, concomitant medications, and any pre-existing clinical symptoms (pre-MDA events) were recorded from all the study participants. For measuring their nutritional status, their weight in kilograms and height in centimeters were converted to the height-for-age Z score (HAZ) and the body mass index (BMI)-for-age Z score (BAZ) using the WHO Anthro-plus software for school-age children [30]. Participants with values less than two standard deviation for both the HAZ and BAZ scores were considered as stunted and wasted, respectively.

### 2.3. Pre-Screening for S. mansoni and STH Infection

Two weeks prior to the MDA, 1484 (25.4%) out of the 5848 enrolled children were pre-screened for *S. mansoni* and STH infections. The number of prescreened children was proportionally distributed across the study schools and districts. The screening for a *S. mansoni* and STH infection was done using the standard Kato–Katz technique recommended by the WHO [31]. In brief, fresh stool samples from each study participant were collected and two Kato–Katz smears were prepared [32]. The egg counts from two smear readings were recorded, and the average value was taken and converted to the eggs per gram of stool using a factor of 24. The intensity of the infection was determined for each parasite as light, moderate, and heavy, based on the WHO criteria.

### 2.4. Mass Drug Administration and Safety Outcome Measures

The study participants received a single dose of praziquantel and albendazole, provided through the school-based MDA campaign led by the district health office of the NTD control program. The praziquantel dose was calculated according to height of the children (≥94 cm dose pole, designed to deliver a dose of at least 40 mg/kg) and 400 mg of albendazole was administered following the national and WHO MDA guidelines [33]. The MDA program was organized and implemented nationwide by the national NTD public health program, and the study team had no role in the implementation of PC. After receiving the MDA, the study participants were actively monitored for any MDA-associated AEs on days 1 and 7 post-MDA. The participants were requested to record any adverse event during days 2–6. Any adverse events reported after receiving the MDA by each study participant were crosschecked and verified to differentiate MDA-associated AEs from any which were previously reported in an event pre-MDA.

The primary study outcome was the incidence of experiencing at least one type of MDA-associated AE (post-MDA AEs), defined as any event that was not reported before receiving the MDA but occurred after the drug exposure. The type and severity of the AEs were secondary outcomes. The severity grading of the treatment-associated adverse events were done using the Common Terminology Criteria for Adverse Events (CTCAE) Version 5.0 [34], as follows:

- Grade 1—Mild: asymptomatic or mild symptoms; clinical or diagnostic observations only; and intervention not indicated.
- Grade 2—Moderate: limiting age-appropriate instrumental activities of daily living (ADL). Minimal, local, or non-invasive intervention indicated.
- Grade 3—Severe: medically significant but not immediately life-threatening: disabling and limiting the self-care activities of daily living. Hospitalization or prolongation of hospitalization indicated.
- Grade 4—Life-threatening consequences: urgent intervention indicated.
- Grade 5—Death related to an AE.

### 2.5. Statistical Analysis

Data were entered on an open-source mobile data-based application Open Data Kit (ODK) for collecting information onto the Ethiopian Food and Drug Authority database and exported to an Excel file for cleaning. The data analysis was done by Statistical Package for Social Sciences (SPSS) version 24. Descriptive statistics was used to analyze the socio-demographic and baseline data. The Chi-square test was used to analyze the associations between the outcome variable (having any AEs or not) with the independent categorical variables. Univariate and multivariate regression analysis using binomial regression was used to determine the predictors of adverse events. Variables with a *p*-value ≤ 0.2 on the univariate analysis were included in the multivariate regression model. For interpretation without changing the estimations, we used a log transformation to change the coefficients into the incidence risk ratio (IRR). Probability values of less than or equal to 0.05 (*p* < 0.05) was considered to be statistically significant.

## 3. Results

### 3.1. Baseline Characteristics of Study Participants

**Figure 1:**
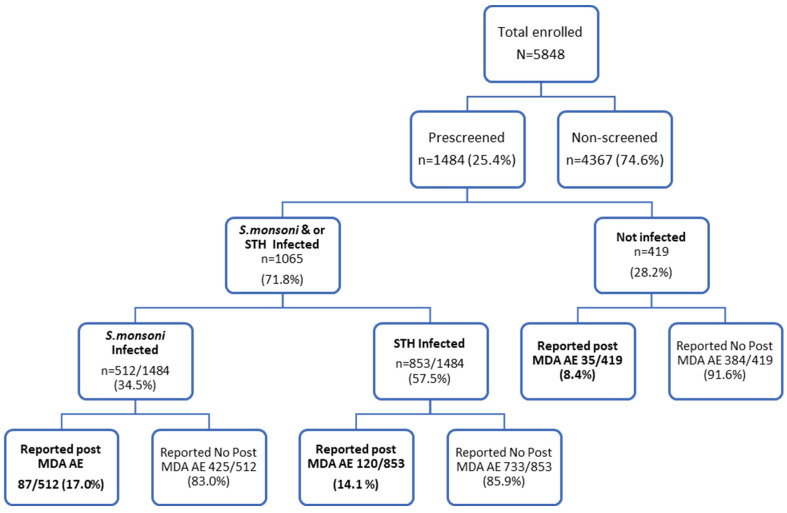
Study flow chart of prescreened participants for *S. mansoni* and soil-transmitted helminth (STH) infections before receiving mass drug administration (MDA) and safety outcomes after MDA stratified by infection status. AEs = adverse events.

A total of 5848 school children (50.5% were male) who were attending six primary schools in the HawellaTulla and Wondo Gennet districts were enrolled in this study. A quarter of the participants, 25.4% (1484), were prescreened for *S. mansoni* and STH infections two weeks before receiving a praziquantel and albendazole MDA. The study flow chart and MDA safety outcomes stratified by parasite infection status is presented in Figure 1.

Among the 1484 prescreened children, 1065 (71.8%) were infected with at least one type of parasite (*S. mansoni* or/and STH). Five hundred and twelve (34.5%) children were *S. mansoni* infected and 853 (57.5%) were STH infected. The baseline characteristics of the study participants are presented in Table 1. Of the total enrolled participants, 2.2% (129) reported pre-MDA events.

### 3.2. Cumulative Incidence of MDA-Associated AEs

**Figure 2:**
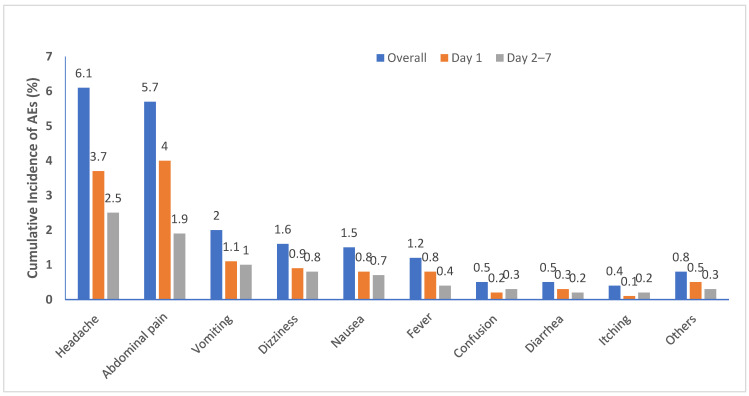
Cumulative incidence of AEs and stratified by days of follow-up.

Thirty-nine (0.7%) out of the 5848 study participants did not complete their day seven follow-up for safety monitoring. A total of 1187 AEs were reported by 780 participants during the seven-day follow-up period. The cumulative incidence of experiencing at least one type of MDA-associated AE during the 7 day follow-up period was 13.3% (780/5848; 95% CI = 12.5–14.2%). Among those who reported MDA-associated AEs, the proportion of individuals who experienced one, two, or three or more types of AEs were 57.7% (450), 34.1% (266), and 8.2% (64), respectively. Of the total participants who experienced at least one type of MDA-associated AE, 63.5% (495) experienced at least one type of AE on day one and the rest, 38.7% (302), experienced an AE within day 2–7 of the follow-up period. The cumulative incidence of AEs over the seven days of the follow-up period stratified by the occurrence days during the follow-up is presented in Figure 2.

### 3.3. Types of MDA-Associated Adverse Events

**Figure 3:**
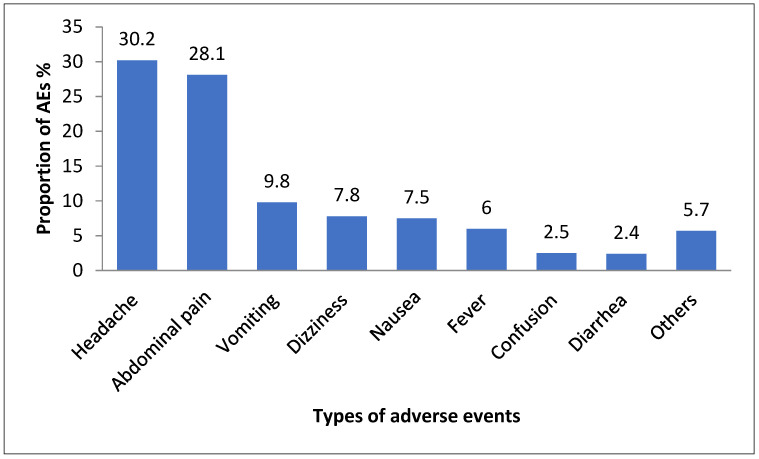
Proportions of AEs stratified by type of AEs.

The most common MDA-associated AEs reported were headache 30.2% (n = 358), abdominal pain 28.1% (n = 334), vomiting 9.8% (n = 116), dizziness 7.8 % (n = 92), and nausea 7.5% (n = 89). The proportion of the adverse events over the seven days of the follow-up stratified by their type of AE is presented in Figure 3. On day one of the follow-up, abdominal pain, at 32.1% (233), was the most reported AE, followed by a headache at 29.5% (n = 214). On the contrary, on day 2–7 of the follow-up period, a headache was the most reported AE, with a proportion of 30.3% (n = 148), followed by abdominal pain, with a proportion of 22.5% (n = 110).

### 3.4. Adverse Events by S. mansoni and STH Infection Status

**Figure 4:**
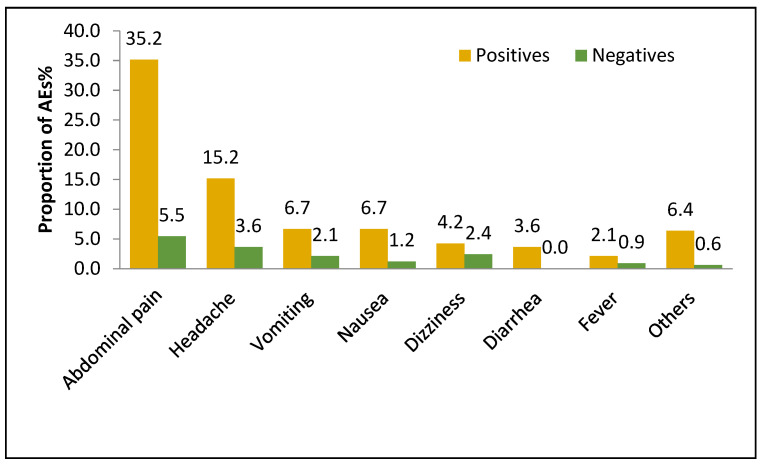
Proportions of AEs among the prescreened participants stratified by *S. mansoni* or STH infection status.

Among the 1484 prescreened participants, 1065 (71.8%) were positive at least for one parasite; out of which, 512 (34.5%) were infected with intestinal schistosomiasis, and 853 (57.5%) were infected with STH. Children infected with at least one type of parasite were more likely to develop MDA-associated adverse events than the non-infected children (15.9% versus 8.4%) (*p <* 0.001). The cumulative incidence of the MDA-associated AEs stratified by the parasite infection status is presented in Figure 1. The cumulative incidence of AEs was significantly influenced by the parasite infection status (*p* < 0.001, χ^2^ = 15.0), with the occurrence of AEs being higher in *S. mansoni*-(17.0%) and STH (14.1%)-infected children than in non-infected children (8.4%). Abdominal pain with an incidence of 35.2% versus 5.5% was the most common AE reported, followed by a headache at 15.2% versus 3.6%, and vomiting at 6.7% versus 2.1% among infected and non-infected children, respectively. The types of AEs stratified by infection status are presented in Figure 4.

### 3.5. Severity Grading of Adverse Events

Out of the total 1187 MDA-associated AEs reported by 780 participants, 85.8% (n = 1019) were mild in their severity grading, followed by moderate at 12.4% (n = 147), and participants with severe AEs were only at an occurrence of 1.8% (n = 21). Most of the AEs were transient and resolved within 2–3 days after the drug administration. No serious AE that required hospitalization (Grade 4) was reported. The summary of the severity grading for each type of AEs is presented in Table 2.

### 3.6. Factors Associated with MDA-Associated Adverse Events

The sex of the participants, their age group, their wasting status, and their parasite infection status were independent significant factors associated with experiencing an AE following the MDA. The cumulative incidence of AEs amongst female participants were significantly higher than amongst male participants (*p* < 0.001). Participants in the age group of 10–15 years old experienced more AEs than younger age participants (5–9 years old) (*p* = 0.002). *S*. *mansoni*- or STH-infected children experienced significantly higher AEs compared to non-infected participants (*p* < 0.001). Children infected with *S*. *mansoni* were more likely to develop AEs than participants infected with STH (*p* < 0.001). The occurrence of AEs was not associated with the stunting status or number of praziquantel tablets taken. The cumulative incidence and factor associations with the MDA-associated adverse events during the follow-up is presented in Table 3.

### 3.7. Predictors of Adverse Events

To identify the factors predicting the occurrence of at least one type of MDA-associated AE, we conducted univariate followed by multivariate log binomial regression analysis. The sex of the participants, their age category, their enrolment site, their wasting status, and their infection types were significant predictors for developing at least one post-MDA AE in the univariate analysis. In the multivariate analysis, their sex, age category, and infection type remained significant predictors of AEs following the MDA; the female sex, participants in the age category of 10–15 years, and participants infected with schistosomiasis had the highest risk of developing AEs post-MDA (Table 4).

## 4. Discussion

A school-based mass praziquantel and albendazole preventive chemotherapy to all at-risk populations without a prior diagnosis has been implemented in schistosomiasis and STH endemic countries for many years. Although safety monitoring in MDA programs is recommended, it is not practiced, especially in SSA countries due to the limited pharmacovigilance capacity. We conducted a large-scale active safety surveillance of AEs following a mass praziquantel and albendazole administration in school children living in high endemic districts of southern Ethiopia. Our study was well controlled to differentiate the MDA-associated adverse events from any pre-existing clinical symptoms. Most previous studies investigated treatment-associated AEs among infected children or MDA-associated AEs in the general population. To investigate whether drug safety is influenced by a helminth infection status, we prescreened one-fourth of the study participants for *S. mansoni* and STH infection. To our knowledge, this is the first large sample size active safety surveillance study to investigate the incidence, types, severity, and risk factors of MDA-associated AEs following praziquantel and albendazole PC considering the participants’ helminth infection status.

The overall cumulative incidence of experiencing at least one type of MDA-associated AE within a week of MDA exposure was 13.3%. The incidence of developing one, two, or three or more types of AEs was 57.7%, 34.1%, and 8.2%, respectively. The most common AEs, in descending order, were a headache, abdominal pain, vomiting, dizziness, and nausea. The participants’ sex, age group, enrolment site, and infection type were significant factors associated with the occurrence of AEs following the mass praziquantel and albendazole administration. Using a similar study design, a recent study from Rwanda reported that one in five child who received a praziquantel and albendazole MDA reported at least one type of MDA-associated AE [35]. The cumulative incidence of an MDA-associated AE (13.3%) in our study was slightly lower than the report from Rwanda, which could be due to variations in the prevalence of an STH or *S. mansoni* infection status of the study participants [32,36]. Using various study designs, follow-up durations, and study populations, incidences of AEs ranging from 8.6% to 83% were reported [21,22,23,37].

Most reported AEs were mild and moderate on the severity grading, transient, and self-limiting, which resolved within 2–3 days of the drug administration. This indicates that PC through a mass praziquantel and albendazole administration is safe and tolerable. Although few severe adverse events were reported (1.8%), none of them required hospitalization and no serious outcome was recorded. Similar observations were reported from other safety studies and meta-analyses [20,22,23,38,39]. Though few, the finding of severe AEs in our study ascertains the importance of integrating active pharmacovigilance in the routine MDA program.

The total number of AEs observed was 1187 from 780 study participants, indicating that some participants experienced more than one type of AE. The occurrence of AEs following the praziquantel and albendazole administration is associated with one’s age, infection intensity, and nutritional status [20,21,22]. In our study, the most frequently reported AEs were headaches, abdominal pain, vomiting, dizziness, and nausea, which is in line with the previous findings [20,38]. Similar AEs were reported to the global pharmacovigilance data base at the WHO-UMC through spontaneous reporting [40]. Most of these AEs are similar to the signs and symptoms of a parasitic infection. However, to differentiate the true AEs associated with the MDA from the parasite infection symptoms, before the MDA, the study participants were interviewed to see if they had any pre-existing clinical symptoms (pre-MDA event) and the participants who reported similar symptoms at the baseline and at the post-MDA follow-up were excluded from the analysis.

The incidence of AEs was significantly higher among female participants compared to male ones (15.4% versus 11.3%). Similar sex difference in the incidence of AEs was reported in the previous studies [35,38,39]. Recent studies conducted on the safety of a MDA for the control and elimination of lymphatic filariasis in east Africa also reported a difference in the incidence of AEs across the sexes [41,42]. An adverse drug reaction (ADR) analysis of half a century data from the global ADR database based on sex differences also reported a higher proportion of ADR among females [43]. A similar finding was reported by the national Pharmacovigilance Centre in the Netherlands from the analysis of their database based on sex [44]. The higher incidence of AEs among females could be explained by their physiologic difference, which may lead to differences in the activity of enzymes for the drug metabolism, resulting in pharmacokinetics and pharmacodynamics deviations [45,46]. However, the sex-dependent pharmacokinetic and pharmacodynamic variations and its role in the occurrence of AEs associated with praziquantel and albendazole MDA remains to be investigated.

We observed a higher incidence of AEs among the older age groups (10–15 years), which is in line with the reported findings from previous studies and meta-analyses [22]. Perhaps this can be explained by the higher dose of praziquantel taken by this age group, as the drug was given based on height. A higher dose of praziquantel is associated with an increased risk of AEs in the previously reported studies [35].

To investigate whether the incidence or type of AEs is influenced by a parasite infection, one fourth of the study participants were pre-screened for *S. mansoni* and STH two weeks prior to receiving the MDA. Interestingly, we found a significantly higher incidence of MDA-associated AEs in children infected with *S. mansoni* and STH than non-infected children. Although a treatment-associated AE is significantly influenced by infections intensity among infected children [21], to our knowledge, ours is the first study to compare the type and incidence of MDA-associated AEs between infected and non-infected children who received a praziquantel and albendazole MDA for PC. Praziquantel-associated AEs in schistosomiasis-infected children may occur due to the drug effect or by the parasite itself. Thus, the reported high AEs among the infected children could be due to dying parasites resulting from the effect of the drugs on the parasites [47]. Further safety surveillance studies which evaluate and compare the difference in the occurrence of AEs among infected and non-infected groups is recommended for supporting the further risk benefit analysis of PC among non-infected individuals, particularly in low endemic areas.

## 5. Conclusions

Preventive chemotherapy with a praziquantel and albendazole combination is generally safe and tolerated by school children. Most of the observed AEs were of a mild to moderate grading and transient, resolving themselves within a week. Being female, older age group (10–15 years), or having a helminth infection are significant independent risk factors for the occurrence of AEs following a praziquantel and albendazole MDA. Though few, the finding of severe AEs and the significantly increased risk of AEs among helminth-infected children underscore the need for the integration of pharmacovigilance in MDA campaigns, especially in high endemic areas for the timely detection and management of AEs and to boost public confidence in the program.

## Figures and Tables

**Table 1:** Socio-demographic and baseline characteristics of study participants.

Variables	Category	Frequency N (%)
Sex	Male	2956 (50.5)
	Female	2892 (49.5)
Age Group	5–9 years	1046 (17.9)
	10–15 years	4802 (82.1)
Enrolment site	Hawella Tula	2306 (39.4)
	Wondo Gennet	3542 (60.6)
Types of infection (n = 1484)	Non-infected	419 (28.2)
	Schistosomiasis only	211 (14.2)
	STH only	552 (37.2)
	Schistosomiasis + STH	302 (20.4)
Stunting status	Normal	5228 (89.4)
	Stunted	620 (10.6)
Wasting status	Normal	5391 (92.2)
	Wasted	457 (7.8)
Type of food eaten Pre MDA.	Carbohydrate	5753 (98.4)
	Fatty meal	50 (0.9)
	Protein	45 (0.8)
Pre MDA-Events	Yes	129 (2.2)
	No	5719 (97.2)

STH = soil transmitted helminths; MDA = mass drug administration.

**Table 2:** Severity grading of AEs after mass praziquantel and albendazole administration.

Types of Adverse Events	Cumulative		Severity Grading		
	Number	Incidence	Mild (N/%)	Moderate (N/%)	Severe (N/%)
Headache	358	6.1	319 (89.1)	35 (9.8)	4 (1.1)
Abdominal pain	334	5.7	294 (88.0)	36 (0.8)	4 (1.2)
Vomiting	116	2.0	88 (75.9)	24 (20.7)	4 (3.4)
Dizziness	92	1.6	80 (87.0)	11 (12.0)	1 (1.1)
Nausea	89	1.5	74 (83.1)	13 (14.6)	2 (2.2)
Fever	71	1.2	58 (81.7)	12 (16.9)	1 (1.4)
Confusion	30	0.5	27 (90.0)	3 (10.0)	0
Diarrhea	29	0.5	20 (69.0)	7 (24.1)	2 (6.9)
Itching	21	0.4	21 (100)	0	0
Drowsiness	13	0.2	13 (100)	0	0
Loss of appetite	10	0.2	5 (50.0)	4 (40.0)	1(10.0)
Rash	4	0.1	4 (100)	0	0
Difficulty Breathing	3	0.1	2 (66.7)	1 (33.3)	0
Cough	1	0	1 (100)	0	0
Other symptoms	16	0.3	13 (81.3)	1 (6.3)	2 (12.5) *
Total	1187	13.3	1019 (85.8)	147 (12.4)	21 (1.8)

* Other symptoms—tremor.

**Table 3:** Cumulative incidence AEs following mass praziquantel and albendazole administration and associated factors.

Variables	Category	Adverse Events		X^2^	*p*-Value
		No (N/%)	Yes (N/%)		
Total		5068 (86.7)	780 (13.3)		
Sex	Male	2622 (88.7)	334 (11.3)	21.5	<0.001
	Female	2446 (84.6)	446 (15.4)		
Age group	5–9 years	938 (89.7)	108 (10.3)	10.0	0.002
	10–15 years	4130 (86.0)	672 (14.0)		
Stunting	Normal	4530 (86.6)	698 (13.4)	0.008	0.93
	Stunted	538 (86.8)	82 (13.2)		
Wasting	Normal	4653 (86.3)	738 (13.7)	7.4	0.007
	Wasted	415 (90.8)	42 (9.2)		
Enrolment district	Hawella Tula	1961 (85.0)	345 (15.0)	8.7	0.003
	WondoGennet	3107 (87.7)	435 (12.3)		
Infection status (*S.mansoni* or STH)	Infected	896 (84.1)	169 (16.0)	14.3	<0.001
	Non-infected	384 (91.6)	35 (8.4)		
Infection types	Non infected	384 (91.6)	35 (8.4)	24.5	<0.001
	Schistosomiasis only	163 (77.3)	48 (22.7)		
	STH only	470 (85.1)	82 (14.9)		
	Schistosomiasis + STH	263 (87.1)	39 (12.9)		
Number of praziquantel tablet taken	<3 tablets	2416 (87.1)	357 (12.9)	1	0.32
	≥3 tablets	2652 (86.2)	423 (13.8)		

STH = Soil-transmitted helminth.

**Table 4:** Predictors of AEs following mass praziquantel and albendazole administration for school children.

Variables	Category	Univariate Analysis			Multivariate Analysis		
		cRR	95% CI	*p*-Value	aRR	95% CI	*p*-Value
Sex	Male	1					
	Female	1.4	1.2–1.6	<0.001	1.3	1.0–1.7	0.04
Age group	5–9 years	1					
	10–15 years	1.4	1.1–1.6	0.002	1.5	1.0–2.1	0.04
Stunting (HAZ)	Normal	1					
	Stunted	1.0	0.8–1.2	0.93			
Wasting (BAZ)	Normal	1					
	Wasted	0.7	0.5–0.9	0.008	1.3	0.8–2.1	0.3
Type of meal eaten before MDA.	Carbohydrate	1					
	Fatty meal	1.2	0.6–2.2	0.6			
	Protein	0.8	0.4–1.9	0.7			
*S.mansoniand*/or STH Infection	Non-Infected	1					
	Infected	1.8	1.3–2.6	<0.001			
Type of Infection	Negatives						
	SM only	2.6	1.8–3.9	<0.001	2.5	1.7–3.7	<0.001
	SM + STH	1.5	0.97–2.3	0.07	1.5	1.2–2.5	0.005
	STH only	1.7	1.2–2.5	0.003	1.7	0.98–2.3	0.057
Number of praziquanltel tablets	<3 tablets						
	≥3 tablets	1.1	0.9–1.6	0.32			

Due to the co-linearity of *S.monsoni* and/or STH infection and type of infection, the variable *S.monsoni* and/or STH Infection was not included in the adjusted model. cRR = crude relative risk, aRR = adjusted relative risk, CI = confidence interval. STH = soil transmitted helminths; MDA = mass drug administration; HAZ = height-for-age z- scores; BAZ = BMI-for-age z-scores.

## Data Availability

All data presented in this study are contained within the manuscript.
